# Experimental study on thermal properties and electrical conductivity of stabilized H_2_O-solar glycol mixture based multi-walled carbon nanotube nanofluids: developing a new correlation

**DOI:** 10.1016/j.heliyon.2019.e02385

**Published:** 2019-08-29

**Authors:** P. Ganesh Kumar, D. Sakthivadivel, M. Meikandan, V.S. Vigneswaran, R. Velraj

**Affiliations:** a Institute for Energy Studies, Department of Mechanical Engineering, CEG, Anna University, Chennai, 600 025, Tamil Nadu, India; b School of Mechanical Engineering (SMEC), Vellore Institute of Technology, Vellore, 632014, Tamil Nadu, India; c Vel Tech Rangarajan Dr. Sagunthala R&D Institute of Science and Technology, Avadi, Chennai, Tamilnadu, India

**Keywords:** Chemical engineering, Nanotechnology, Viscosity, Multi-walled carbon nanotubes, Thermal conductivity, Electrical conductivity, Solar glycol, Density, Rheology, Heat transfer, Nanofluidics, Nanoparticles, Carbon nanotubes

## Abstract

The main aim of this present work is to explore the influence of dispersion of MWCNTs in a mixture of water-solar glycol (70:30) on its electrical conductivity and thermophysical properties such as density, rheology and thermal conductivity. The MWCNTs were seeded with a various weight percentage of 0.15, 0.3 and 0.45 via a common two-stage synthesis technique. The homogeneous stability of MWCNTs based nanofluids was confirmed by high-resolution scanning electron microscopy and ultraviolet-visible spectroscopy. The density of solar glycol and H_2_O mixture based MWCNTs nanofluids were measured with standard borosil volumetric flask via weighing balance mechanism and the experimental findings displayed a good agreement with the well-known correlation of Pak and Cho owing to the natural packing of H_2_O inside the nanomaterial in a limited quantity. The thermal conductivity of 0.45 wt. % MWCNTs seeding got augmented by 19.12% at ambient temperature while the electrical conductivity got augmented by 93.54% at 50 °C. Therefore, the augmentation in the thermal conductivity of water/solar glycol mixture with 0.45 wt. % MWCNTs seeding is because of the kinetics of nanomaterial accumulation and fluid layering. In addition, mathematical correlations were recommended for estimating the ratio of the thermal conductivity and viscosity of the nanofluid at different weight fractions.

## 1 Introduction

In this century, nanofluids have fascinated a great interest among the research community and various industrial applications such as chemical, solar thermal, transportation, microelectronic, refrigeration and air-conditioning, power plants, petrochemical, and defense [1, 2]. The thermodynamic properties of HTFs such as viscosity, thermal conductivity (k), density and dielectric constant act as the most influencing properties to be taken into account while developing an energy-efficient heat transfer equipment [3, 4]. In the future, there is an enormous opportunity in the current and forthcoming years to enrich the energy efficiency (ղ) and a heat transfer rate of the solar and other thermal systems. The performance of the heat exchange mechanisms improves with the incorporation of energy-efficient mechanism in it, but these benefits are huge when it is incorporated in heat exchangers. These benefits not only help to bring down the cost associated with thermal energy systems but also helps to mitigate the emission of greenhouse gases thereby reducing the effect of global warming considerably [5]. The heat exchange performance of the solar thermal system, electronic cooling system, heat recovery systems is generally reliant on the heat transfer properties of the operating fluid normally mentioned to as Heat Transfer Fluids (HTFs). Some of the prominent HTFs are H_2_O [6, 7], ethylene glycol [8, 9], solar glycol [10, 11, 12, 13, 14], propylene glycol, bio glycol [15], ionic liquid [16] and air. Tremendous hard works were attempting by numerous industrialist, scientist and researchers to upsurge the heat transfer properties of the operating fluids through the scattering of nanomaterials [17, 18, 19] holding high thermal conductivity value. Nevertheless, the difficulties are noticeable like rapid settlement of seeded nanomaterials blocking the flow channel, erosion, and corrosion, which restrictions its use in heat exchange. To overwhelmed the above-stated challenges, Choi et al. [20] proposed a pioneering way of improving the heat transfer properties of the base fluid by the uniform seeding of high conduction nanomaterials at nanoparticle measure level called as nanofluids. These interesting fluids possess higher thermal transportation properties associated with the base fluids even when added at a lower nanomaterial concentration [21]. The Multi-Walled Carbon Nanotubes (MWCNTs) are straight fibrous morphology tubes with remarkably good electrical, thermal and mechanical properties; they have pinched the attention of different scientist/researchers to make and detect good operating fluids in various mixtures of the base fluids. Meanwhile, the lower density of carbon-fiber-based nanofluids helps in the easy synthesis of well-seeded energetic HTF without any most important limitations namely nanomaterial settlement in a minimum duration. Important scientific/research studies have been done to augment the thermal properties of single-wall (SW) and multi-wall (MW) carbon-based nanofluids as summarized below:

Jayabalan Ganeshkumar et al. [22] experimentally measured the density of MWCNTs based nanofluids using two different volumetric container technique and their research data exposed a great accord with the Pak and Cho equations. Ganesh Kumar et al. [12] experimentally studied the SG based nanofluids comprising MWCNTs nanomaterials with various nanomaterial concentrations for instance 0.1 vol. %; 0.2 vol. %; 0.3 vol. % and 0.4 vol. %. From the results, it was noticed that the nanofluid density rises with the increase in CNT mass percentage. This is because of the formless and shapeless combination of nanofibers with SG in a confined style, which in turn, rises the mass of the nanofluids for a well-known bulk volume.

The thermal conductivity (k) of the nanofluids is the most significant factor, which influences the percentage increase of the convective heat transfer coefficient (CHTC) [23]. There are numerous parameters that influence the “k” of nanofluid for instance (a) concentration, (b) size and shape of the nanomaterial (c) temperature, (d) homogeneous stability of nanofluids and (e) surface to volume ratio of nanomaterials [24, 25, 26, 27]. Hwang et al. [28] experimentally exposed the “k” of several types and shapes of nanomaterials such as MWCNTs, SiO_2,_ and CuO seeded in EG and water-based fluids. Their experiential result showed that the positive tendency of thermal conductivity as the temperature rises. Timofeeva et al. [29] experimentally found that better enhancement in thermal conductivity was achieved in EG-based nanofluids when compared to water-based nanofluids. Very few researchers and scientists have focused on MWCNTs based nanofluids to enhance its thermal conductivity as presented in Table 1.

**Table 1:** Experimental studies in thermal conductivity of MWCNTs based nanofluids.

Author Name	Base-fluid	Concentration range	Inference
Ganeshkumar et al. [22]	Water-EG mixture	0.3, 0.6, 0.9, and 1.5 wt. %	The thermal conductivity improvement of 11% was achieved when the nanofluids holding 0.9 wt.% of MWCNTs.
Mansoor Farbod et al. [30]	Deionized water	CNT based nanofluids were seeded with 0.1, 0.25 and 0.5 vol. % of the pristine, 1, 2, and 4 h refluxed CNTs to demineralized water.	The “k” of nanofluids increases with lessening the length of the CNTs, and with increasing the temperature.
Hwang et al. [31]	Mineral oil	0.5 vol.%	MWCNT/oil-based nanofluid showed poor stability as compared to other nanofluids
Meibo Xing et al. [32]	De-ionized water	0.05, 0.14, 0.24, 0.33 and 0.48 vol. %. Temperature Range 10–60 °C	The thermal conductivities of the nanofluids rises with an increase in temperature and the volume concentration. Long single-walled nanotubes (SWCNT) based nanofluids having the utmost thermal conductivity value.
Munkhbayar et al. [33]	Distilled water	Grinding Speed 300,400,500 and 600 rpm.	The raw MWCNTs nanofluid prepared at a grinding speed of 600 rpm achieved a maximum thermal conductivity enhancement of 18.12%.
Ganesh Kumar et al. [12]	Solar Glycol	0.1%, 0.2%, 0.3%, and 0.4% vol. %.	The thermal conductivity (*k*) of the MWCNTs based nanofluids improved by approximately 17.3% at the volume fraction of 0.4 at 70 °C.

White et al. [34] achieved augmentation in the Electrical Conductivity (EC) by 100 times with the seeding of 7 vol. % of nanomaterials. It was also detected that the EC augmented with a reduction in the size of the nanomaterial. Ganguly et al. [35] conveyed that EC is influenced by several parameters for instance agglomeration, stability, and Brownian motion. Shen et al. [36] selected insulated oil as the base fluid and ZnO as a nanomaterial. They observed 973 times augmentation in the EC at 0.75% volume concentration. S. Nabati Shoghl et al. [37] experimentally computed the thermal properties of six different H_2_O based nanofluids as functions of volume fraction and various temperature. The dynamic viscosity (μ) of the nanofluid good agreement with the Einstein model in lower weight fractions while as the fractions increased, the viscosity forecast using this model was lower than the measured viscosity that may be capable to the variations in the performance of the nanofluid. Ganesh Kumar et al. [38] experimentally investigated the thermal conductivity and EC enhancement of SG-water (50:50) mixture containing MWCNTs. The electrical conductivity augmentation of nearly 62.19% was achieved for 0.6 vol. % of MWCNTs in water - SG (50:50) mixture at 50 °C. A few types of research as presented in Table 2 studied the viscosity of MWCNTs based nanofluids.

**Table 2:** Some of the research works focused on the viscosity of MWCNTs based nanofluids.

Author Name	Base-fluid	Concentration range	Inference
Chen and Xie [39]	Silicon oil	0.2–1 vol. %	∼30% enhancement at 1 vol. %
Fontes et al. [40]	Transformer oil	0.005–0.05 vol. %	25% enhancement at 0.05 vol. %
Lifei Chen et al. [39]	Water, ethylene glycol, glycerol, and silicone oil		The viscosity almost remained constant when the temperature exceeds 55 °C.
Mohammad Hemmat Esfe [41]	Engine Oil (10 W40)	0.025, 0.05, 0.1, 0.25, 0.5,0.75 % Volume fraction	The apparent viscosity and consistency index of nanofluid rises with increase in nanomaterial volume fraction.
Ding et al [42]	Water	0.1–0.5 wt. %	The viscosity of nanofluids rises with an increase in CNT concentration and decreasing temperature
Paritosh Garg et al. [43]	DI water	1 wt. % MWCNT, 0.25 wt. % GA, ultra-sonicated for 20, 40, 60 and 80 min.	The viscosity of the nanofluids rises with respect to sonication time.
Ganeshkumar et al. [22]	Water-EG mixture	0.3, 0.6, 0.9, and 1.5 wt. %	The viscosity ratio increased by 2.25-fold, for the most part in the temperature range of 30–40 °C.
Ganesh Kumar et al. [12]	Solar Glycol	0.1, 0.2, 0.3 and 0.4 vol. %.	They developed the correlations from the measured value of CNTs based nanofluids, which takes into account base fluid, fluid temperature and volume concentration of nanomaterials. These innovative models effective for (i) nanofluids temperature range of 30–70 °C and (ii) CNT volume fraction range between 0.1 and 0.4 vol. %.

Based on the available literature, only a few research studies were focused on electrical conductivity density, viscosity, and thermal conductivity of SG/H_2_O mixture seeded with MWCNTs under different weight fractions. Therefore, the current experimental work aims to identify the influence of MWCNTs nanomaterials in augmenting the heat transfer properties of SG/H_2_O mixture based nanofluids at altered weight fractions, and the experimental outcomes were related through the existing correlations for heat transfer applications.

## 2 Materials and methods

This subsection deals with broad information about the materials and instruments used for describing the SG/H_2_O mixture based MWCNTs nanofluids and followed by a detailed study of the homogenous stability of the nanofluids.

### 2.1 Materials

This research work, a blend of 30 vol. % SG and 70 vol. % de-mineralized (DI) water has been used as the base fluid while gum arabic or gum acacia (GA) acts as a surfactant and MWCNTs were dispersed in the base fluid to make the nanofluids. The nanomaterial (MWCNTs) was obtained from Mr. Cheap Tubes, the United States of America and the technical stipulations of the CNTs delivered by the enterprise are specified in Table 3.

**Table 3:** Technical stipulations of the MWCNTs.

Descriptions	Stipulations
Length of the tube	10–20 μm
Diameter of outer tube	30–50 nm
Diameter of inner tube	5–10 nm
Area of surface	60 m^2^ g ^−1^
True density	∼2100 kg cm^−3^
Bulk density	∼280 kg cm^−3^
No. of walls	3 to 15
Purity	∼95 wt. %
Appearance	Black

The SG were procured from Dynalene, USA. The chemical and physical properties of SG delivered by the industrialist are revealed in Table 4. The advantages of SG comprise good thermal stability, lower viscosity, harmless and renewable.

**Table 4:** Chemical and Physical criteria of SG.

Parameter		Stipulations
pH value		7.0–11.0
Odor		Odorless
Phase change temperature (Liquid to Gas)		>100 °C (>212°F)
Relative density		1000–1200 kg cm^−3^
Vapor pressure		0.008 cmHg at 25 °C
Dynamic viscosity		>1.0 cP at 25 °C
Appearance		Clear
State		liquid

### 2.2 Preparation of H_2_O/SG/MWCNTs based nanofluids

**Fig. 1:**
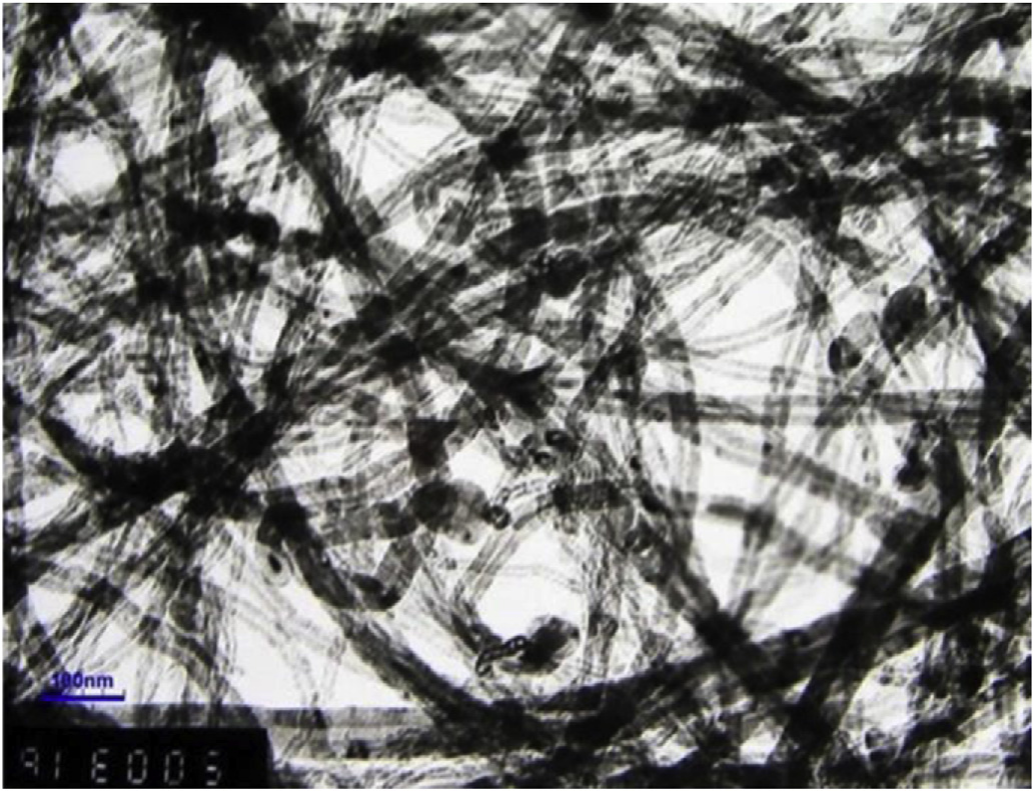
High-Resolution TEM picture of MWCNTs (Source File: Mr. Cheap Tubes Inc., United States),

Three different types of nanofluid samples were equipped with the loading of 0.15 wt. %, 0.30 wt. % and 0.45 wt. % MWCNTs accompanied by 0.25% weight fraction of the GA surfactant for every fluid sample. Fig. 1 displays the Transmission Electron Microscope (TEM) picture of the nanomaterial, and the existence of entanglements is detected in some areas of the picture. In order to overcome this problem, the high-energy nano grinding mill of the CNT nanomaterial was carried out with 1 cm Tungsten balls for 1800 s, followed by ultrasonication process for 1 h under the condition of dry environment.

**Fig. 2:**
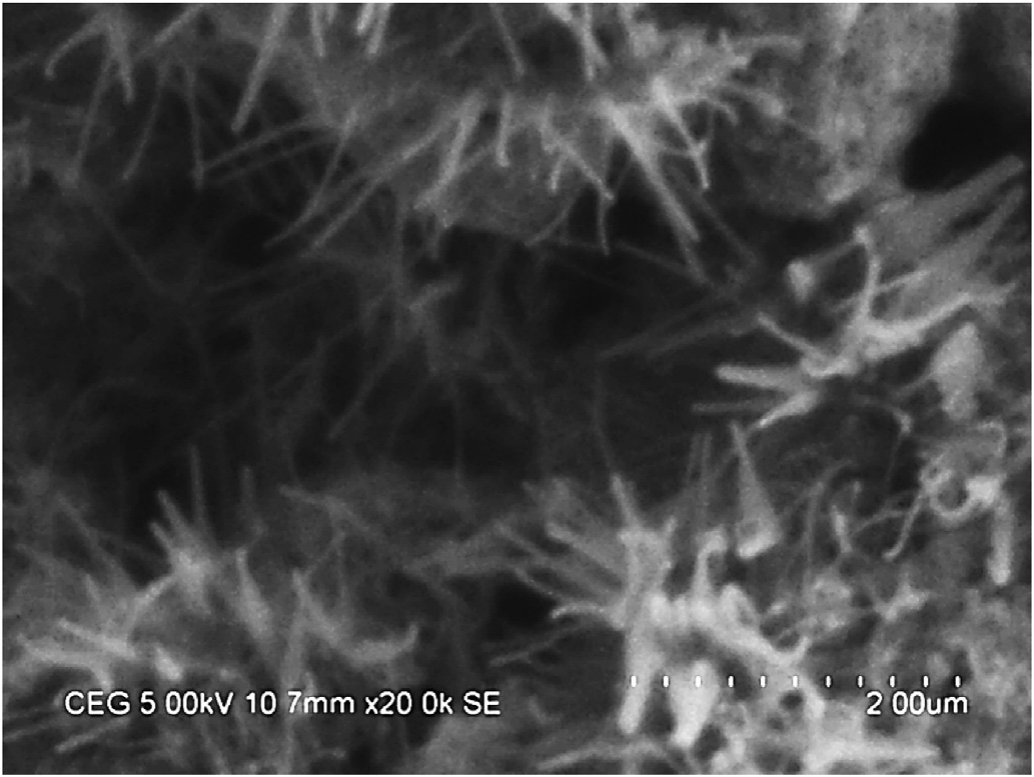
High-Resolution SEM picture of MWCNTs seeded in the H_2_O/SG mixture (0.15 wt. %).

**Fig. 3:**
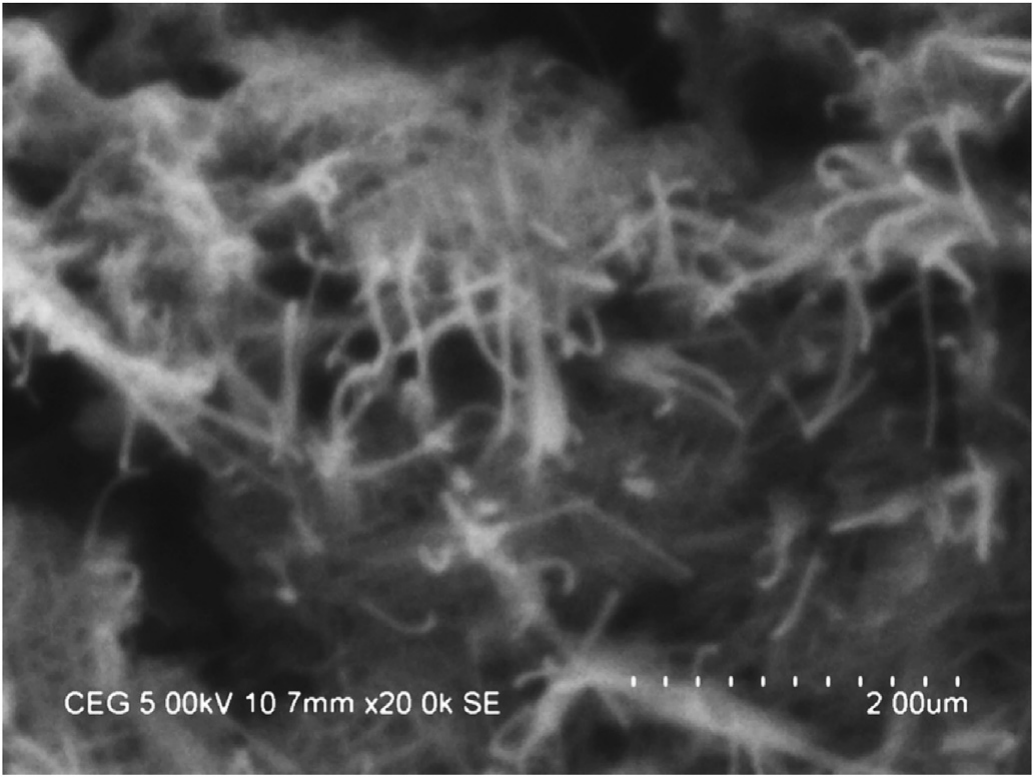
High-Resolution SEM picture of MWCNT seeded in the H_2_O/SG mixture (0.45 wt. %).

The GA surfactant or acacia gum is dissolved in SG and H_2_O blend using **REMI** magnetic mixer or magnetic stirrer (220–240 V, 50 Hz, and 1200 rpm), after that addition of nanomaterial. The fluid solution is continuously stimulated for 45 min after that ultrasonication process for 1 h to confirm the proper distribution of the MWCNT in H_2_O/SG blend. Figs. 2 and 3 show the high-resolution SEM [44, 45, 46, 47] picture of the MWCNTs at different weight fractions seeded in the SG – H_2_O blend nanofluid and the mean diameter of the seeded nanofiber is noticed to be ∼30–40 nm.

### 2.3 Stability of nanofluids

**Fig. 4:**
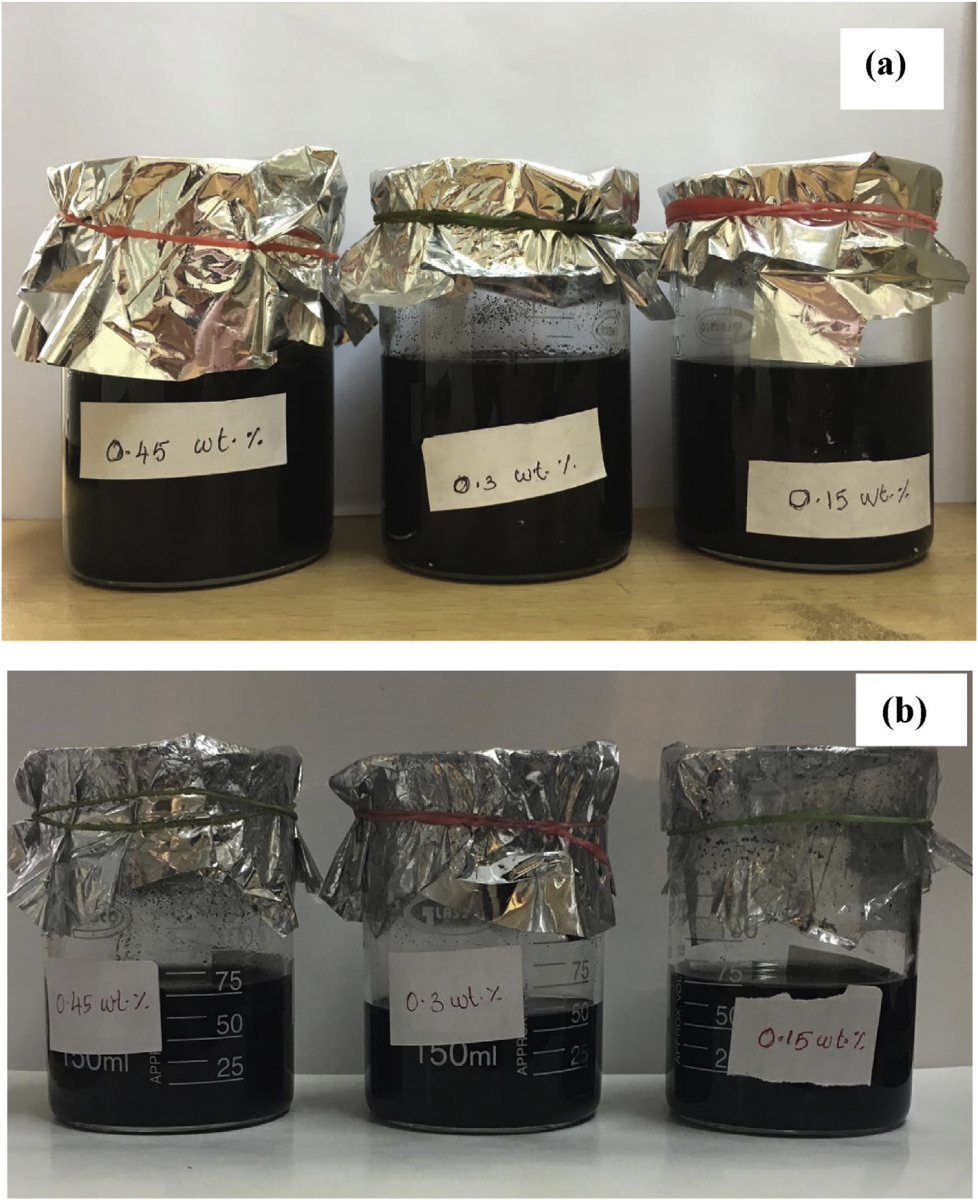
Photographic representation of the nanofluids: (a) after preparation (b) after two months from preparation.

The homogenous stability was additionally confirmed by using UV–Vis study. Fig. 4 (a) and (b) shows the nanofluid at the time of preparation and two months after preparation, respectively. It is observed from the figure, no change of color can be perceived in the nanofluid, it seems like to be more stable for 60 days, and no visible sedimentation was observed. After confirming the homogenous stability of nanofluid, the thermal transport properties of the H_2_O/SG mixture based nanofluid were measured.

### 2.4 Thermal transport characteristics of the nanofluids

#### 2.4.1 Density measurement

Pak and Cho's model was used [48] to estimate the density of nanofluids, which is established on the mixing theory given by,

(1) *ρ*_*nf*_*= ϕ ρ*_*p*_*+ (1- ϕ) ρ*_*bf*_

In this research, the density of the virgin base fluid and MWCNTs based nanofluid was measured at the ambient temperature with the borosil standard volumetric flask (SVF) technique. Two different kinds of borosil class-A SVF of 10 mL and 25 mL were used for calculating the density with minimal uncertainty. Before the taking readings of density, both the SVF was calibrated by using the de-ionized water and pure base fluid with the experimental temperature conditions.

The higher uncertainty included in the density measurement was ±0.3% for 25 ml standard volumetric flask and ±0.6% for 10 ml flask respectively. An identical mass of CNT nanofluid was taken in the borosil SVF and a highly precise digital electronic weighing machine was used to measure the value of the mass of the fluid with an accuracy value of ±0.2% gram. Each sample of the MWCNTs nanomaterial-based stable nanofluids, the readings were noticed for 5 times to make sure the accuracy and repeatability of the measured value. The mean of the measured nanofluid readings was taken, and these mean experimental readings were used for determining the fluid density by using Eq. (2):

(2) $$\rho_{\mathrm{nf}}\text{ ​}=\frac{\left(m_{tf}-m_{f}\right)}{V_{nf}}$$

#### 2.4.2 Measurement of viscosity

An experimental investigation of the rheological behavior of the MWCNTs based nanofluids is made via a CVO-50 shear rate and yield stress digital Rheometer from (Bohlin Instruments Ltd., UK) in the fluid temperature range between 30-50 °C, with a phase of 5 °C. The desired temperature of the SG – water mixture based nanofluid small amount was kept up with the aid of a Peltier cooler and the outward coolant removed the heat produced from the Peltier cooler away. Before the readings, the experimental setup was calibrated by standard Brookfield fluids to confirm the degree of accuracy of the readings. The SG – water blend and nanofluid were present at the bottom disc of the digital rheometer arrangement, ensure that no air balloon was entrapped in the base fluid and nanofluid container. The investigation was performed using automatic micro-controlled at a variable shear rate with the shear stress ranging from 0-10 N m^−2^. In this experimental study, the contour profile of the upper disc was a parallel plate shape with a radius of 2 cm. The linear gap of 70× 10^−6^ m was maintained between the upper and lower disc.

The measured values of SG-water mixture based nanofluids were then compared with those achieved from the existing well-known correlations (Batchelor [49], Drew and Passman [50], Timefeeva et al. [51]) as computed in models 3 to 5.

(3) *μ*_*nf*_ = (1 + 2.5*ϕ* + 6.2*ϕ*^2^) *μ*_*bf*_

(4) *μ*_*nf*_*= (*1 + 2.5*ϕ) μ*_*bf*_

(5) *μ*_*nf*_*= (*1 + 13.5*ϕ +* 904.4*ϕ*^*2*^*) μ*_*bf*_

#### 2.4.3 Thermal conductivity measurement

The Decagon KD2 Pro thermal properties device (an analyzer) is the typical technique for determining the thermal conductivity (*k*) and resistivity of the grained powder and liquid phase sample, which functioning on the fundamental principle operations of the standard transient dynamic technique. The device has a single metal needle sensor with 0.13 cm diameter and 6 cm length, which integrates a thermal resistor and heating element in its inner side. This measurement system is coupled with a microprocessor and microcontroller unit for regulating and conducting the measurements. Decagon KD2 Pro thermal property analyzer is calibrated with demineralized water and SG mixture. The “k” value of H_2_O/SG blend and MWCNTs based nanofluids are measured at atmospheric temperature. For agreeable experimental results, five readings were taken for each sample.

The experimentally measured data's of SG – water mixture nanofluid values were compared with the following present thermal conductivity correlations such as Hamilton and Crosser [52], Wasp [53], Yu and Choi [54], and Timefeeva [55] as computed in models 6 to 9.

(6) $$k_{nf}=\left[\frac{k_{p}\;+\;\left(n-1\right)k_{w}\;-\left(n-1\right)\emptyset \left(k_{w}-k_{p}\right)}{k_{p}+\left(n-1\right)k_{w}+\emptyset \left(k_{w}+k_{p}\right)}\right]k_{bf}$$

(7) $$k_{nf}=\left[\frac{k_{p}\;+\;2k_{w}\;-2\emptyset \left(k_{w}-k_{p}\right)}{k_{p}+2k_{w}+\emptyset \left(k_{w}+k_{p}\right)}\right]k_{bf}$$

(8) $$k_{nf}=\;\left[\frac{\;\;2\left(k_{p}-k_{w}\right){\left(1+\beta \right)}^{3}\phi +k_{p}+\;2k_{w}}{{k_{p}+2k_{w}-\left(k_{p}-k_{w}\right)\;\left(1+\beta \right)}^{3}\phi }\right]k_{bf}$$

(9) $$k_{nf}\;=\;\left(3\emptyset +1\right)k_{bf}$$

### 2.5 Electrical conductivity (EC) measurement

EC of H_2_O/SG blend based MWCNTs nanofluids were calculated through 4-Cell conductivity meter (PICO+) coupled with advanced microcontroller and automatic temperature compensation with range 0°–150 °C. The digital EC meter is calibrated using de-mineralized water (70%) and SG (30%) blend and the consistency of experimental readings are tested through repeated measurement values and is establish to agree well with the standard data. The EC of de-mineralized water and pure solar glycol blend and CNT nanofluids with 0.25 weight fractions of GA is measured at temperatures ranging from 30 to 50 ^ο^C in steps of 5 ^ο^C. For satisfactory experimental outcomes, five readings are taken for each temperature.

## 3 Results and discussion

### 3.1 Stability of H_2_O/SG blend MWCNTs based nanofluids

One of the key parameters that choose the use of the nanofluids as the HTFs is the long-duration homogeneous stability of the seeded nanomaterials in the base fluid. Henceforth, the homogeneous stability of the nanofluid was confirmed by using Ultraviolet-Visible spectroscopy analysis (UV/Vis) analysis.

#### 3.1.1 Ultraviolet-visible spectroscopy analysis

**Fig. 5:**
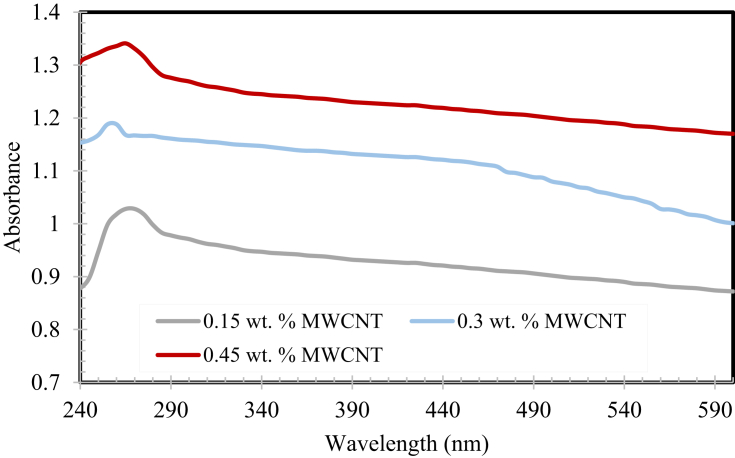
UV/Vis absorbance of MWCNTs based nanofluids at different weight fractions.

The absorbance spectra of MWCNTs based nanofluids at various weight fractions are revealed in Fig. 5. The UV/Vis absorbance at a wavelength of approximately 270 nm for entire MWCNTs based nanofluids samples were calculated experimentally for half months with steps of 3 days for various weight fractions. It can be inferred from the graph that there was a consequent increase in light absorbance with the rise in weight fractions of the nanofluid sample. This could be due to the linear relationship that exists between the weight fractions of fluid and UV/Vis absorbance [56].

**Fig. 6:**
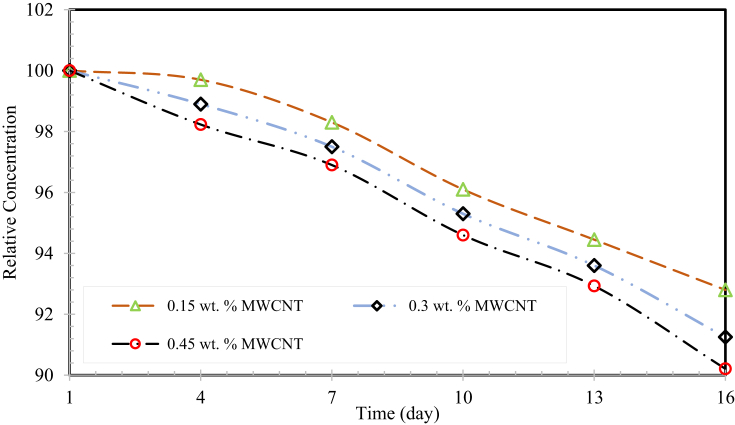
Relative supernatant weight fractions of nanofluids with sediment time.

Fig. 6 clarifies the homogenous stability of MWCNTs in H_2_O and SG mixture. This graph confirms the relative supernatant material weight fraction in an H_2_O and SG blend with settlement time, it was determined by UV/Vis and it's working under the principle of the law of Beer–Lambert's. The UV/Vis absorbance of a CNTs based nanofluids is a direct relationship with weight fractions of the absorbing manner namely CNTs in the base fluid. The relative concentration is defined as the ratio of consequent weight fraction of the nanofluid samples to that of the new one; it was computed for all the liquid samples. It is observed in Fig. 8 that the maximum settlement value of 9.79% was achieved in 16 days for the weight fraction of 0.45 wt. %, which confirmed the appropriate seeding of MWCNTs in the base fluid.

**Fig. 7:**
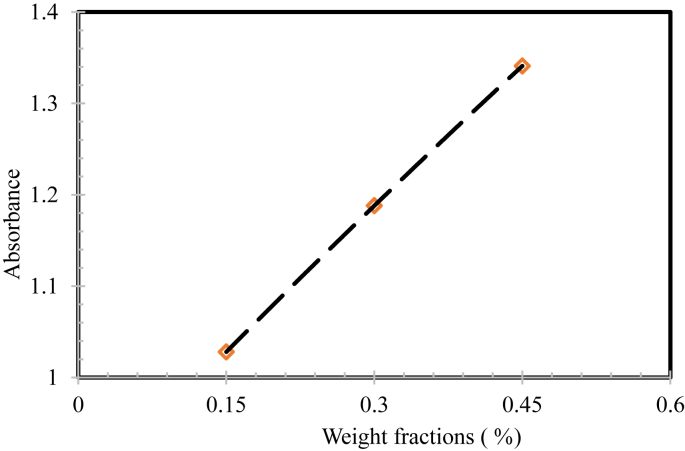
A linear relationship between light absorbance and weight fractions of nanofluids.

Fig. 7 validates that the linear relation was conquered between the weight fractions and the light absorbance of seeded nanomaterials. From these relationships, the relative homogenous stability of CNTs based nanofluids could be evaluated with sedimentation time.

**Fig. 8:**
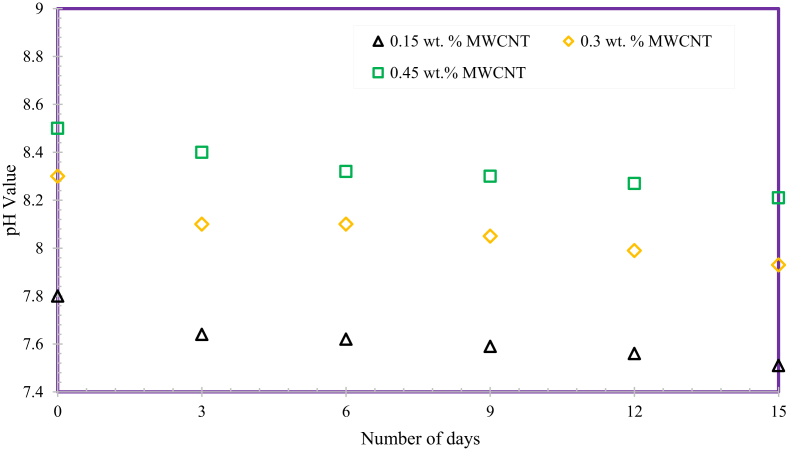
pH variation relating to time.

The pH (the H^+^ ion concentration of a solution) of MWCNTs based nanofluids is the main factor to confirm the homogenous consistency of nanofluids. Fig. 8 indicates that the pH value of entire nanofluid weight fractions diminutions with a rise in days. However, the variation in pH value amongst the first day and fifteenth day is negligible.

### 3.2 Nanofluids thermal characteristics

The various thermal characteristics of MWCNTs/H_2_O and SG mixture based nanofluids are experimentally measured and presented in this section.

#### 3.2.1 Nanofluids density

**Fig. 9:**
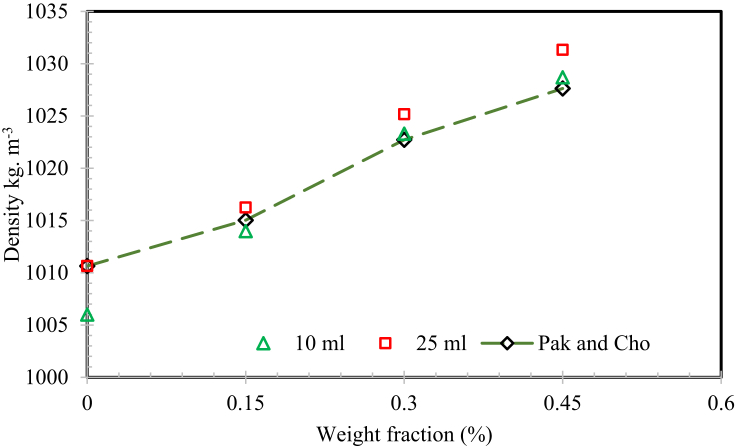
Weight fraction dependence of nanofluid density.

**Fig. 10:**
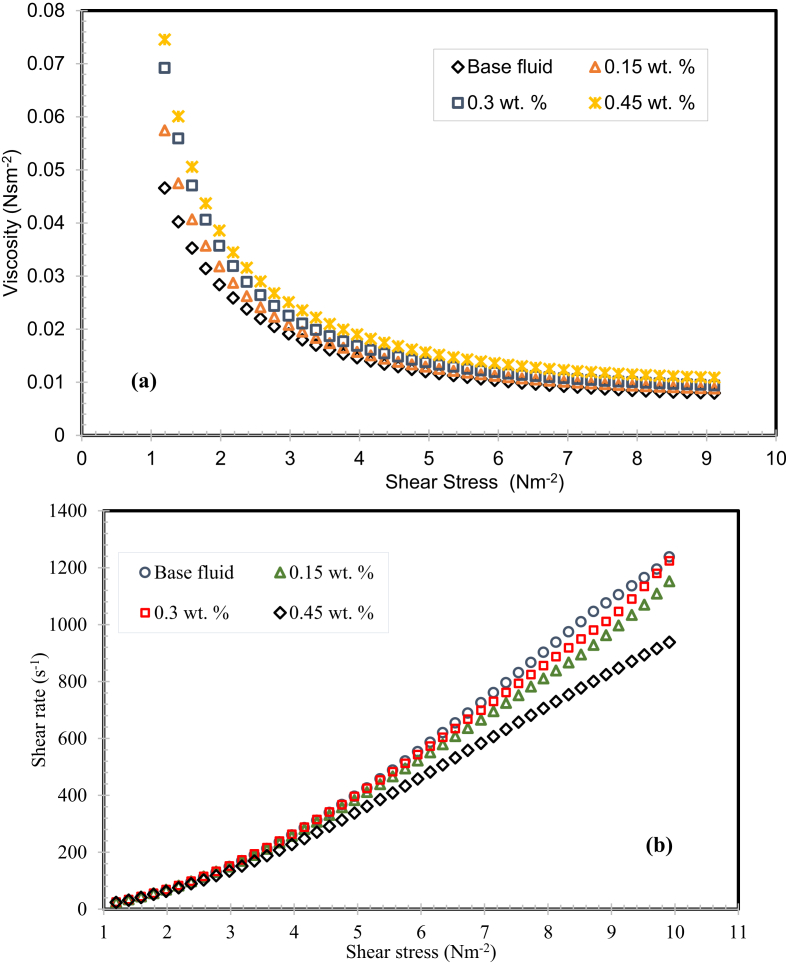
(a) and (b). Rheological characteristics of the nanofluids (T = 40 °C).

The comparison between measured nanofluid density and the estimated nanofluids density which was arrived using Eq. (1) is shown in Fig. 9. The density of the virgin base fluid and nanofluid were estimated as 1009 and 2100 kg m^−3^ respectively. It can be noticed that the nanofluid density upsurges with an increase in the fractions of MWCNTs compared to H_2_O and SG mixture. The measured value presenting a good agreement with the well-known Pak and Cho model data using Eq. (1). It is inferred from the graph that the correlation under forecasts the density by 2.62 and 3.61 kg m^−3^, by using 10 mL and 25-milliliters borosil SVK respectively, when the nanofluid comprises 0.45 wt. % of MWCNTs, owing to promising dispersing of the SG and H_2_O mixture materials with the MWCNTs. It is decided from the computer simulation technique that the pure CNTs is employed with the particles of the virgin base fluid in a various manner namely, layer manner, bulk manner and wire manner [21] (see Fig. 10).

#### 3.2.2 Rheological characteristics of nanofluids

The rheological characteristics of the MWCNTs nanofluids with the different weight fractions of is displayed in image 10 (a) and 10 (b) at 40 °C. From the graph, it can be detected that the rheological characteristics of nanofluid are powerfully reliant on the weight concentration of the nanomaterials. It was found that the dynamic viscosity of H_2_O/SG blend based nanofluid diminishes noticeably with increment in shear stress that evidently designates the non-Newtonian behavior of the nanofluids. The non-Newtonian behavior is mightily stimulated by the weight fraction of MWCNTs in the nanofluid and this section stretched with the rise in weight fraction of the MWCNTs as illustrated in image 10 (a).

The transition section from the non-Newtonian behavior to Newtonian was in the shear stress range of 1–2 Nm^−2^ for the nanofluids comprising 0.15 & 0.3 weight percentage of MWCNTs. Though, this thin and very irregular layer was stretched to a maximum shear stress range of 4–7 N m^−2^ at the weight fractions of the MWCNTs (0.3 & 0.45 wt. %). This is owing to the maximum resistive energy obtainable by the bands of MWCNTs and material-to-material interfaces in the SG – water mixture. These bands start to rearrange with the rise in the shear stress and the nanofluids displayed a close identical dynamic viscosity was achieved after the transition region, which evidently specifies the Newtonian actions as indicated in the graph 10 (b). The same results of rheological characteristics were also conveyed for the nanofluids with the seeded of MWCNTs in water – EG mixture [22].

**Fig. 11:**
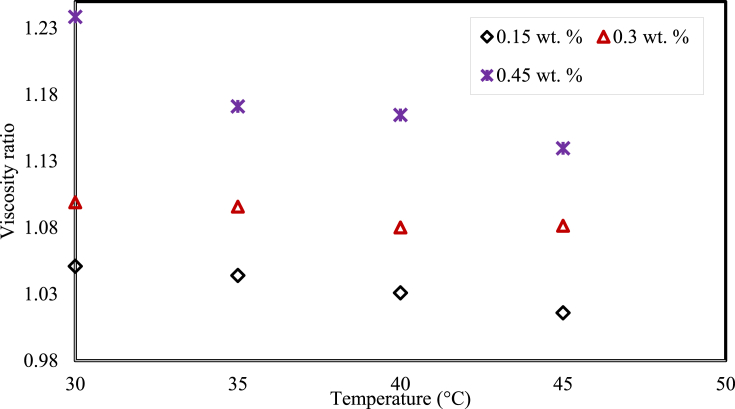
Viscosity ratio of the nanofluid with the effect of temperature.

Fig. 11 displays the viscosity ratio of the MWCNTs based nanofluids in the Newtonian region, at weight fractions with respect to the various temperatures of MWCNTs. It can be observed that the viscosity ratio diminishes with an increment in the nanofluid temperatures. The nanofluid with 0.45 wt. % showed enhancement in dynamic viscosity by 1.24 times when compared to the dynamic viscosity of the virgin base fluid at 30 °C.

**Fig. 12:**
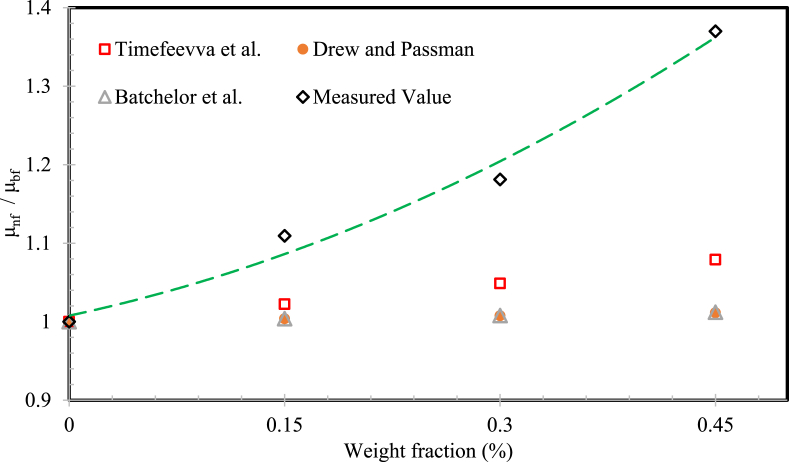
Viscosity ratio comparison with several correlations (T = 40 °C).

The measured experimental results from this research study were compared with the theoretical correlations offered by Batchelor et al. [49], Draw and Passman [50] and Timofeeva et al. [51], at a temperature of 40 °C as shown in Fig. 12. The measured values of SG-water mixture based nanofluids at various weight fraction are higher compared with the theoretical correlations considered. The compared data's absolutely demonstrations that the experimental data are significantly maximum than the calculated values for all the nanofluids. This was because of the variance in particle size, preparation technique, nanomaterial scattering, nanoparticle cluster and interfacial layer between nanomaterial and glycol and water mixture. Henceforth, the models for predicting the viscosity ratio of the MWCNTs-SG/water mixture based nanofluids has been developed in the following formula:

$$\text{Viscosity ​ratio}{(\mu }_{r}\text{)}=\frac{\mu_{nf}}{\mu_{bf}}\text{ ​}=\text{ ​}0.8834\phi^{2}\text{ ​}+\text{ ​}0.3903\phi \text{ ​}+\text{ ​}1.0077$$

#### 3.2.3 Thermal conductivity of nanofluids

**Fig. 13:**
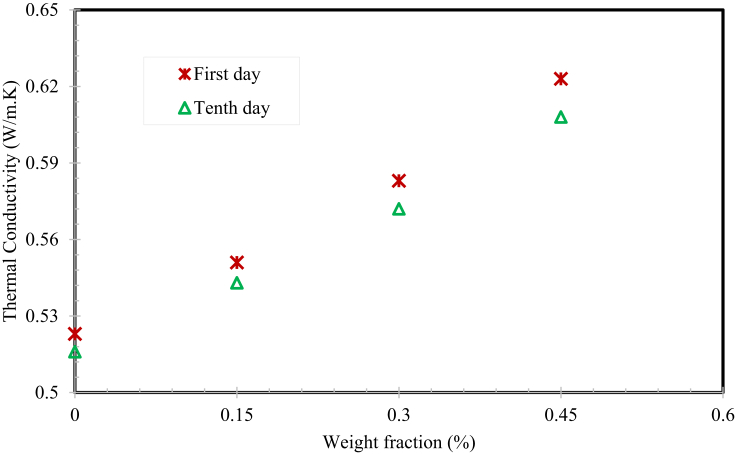
Nanofluids thermal conductivity at room temperature.

Fig. 13 displays, the thermal conductivity of MWCNTS/water – SG mixture based nanofluids measured on the first and tenth day after preparation. It is approved that the prepared CNTs/water and SG mixture based nanofluids are homogenously stable and the usage of CNTs based nanofluids is suitable for different convective heat transfer applications for better performance. The “*k”* of MWCNTs and water/SG based nanofluids increase linearly with nanomaterial weight fraction. The figure confirmations that the “*k” value* increased with increase in nanomaterial weight loading. This happened because the nanomaterials had maximum particle surface area to volume ratio. The augmentation mechanism might be owing to the material-to-material interface, nanoparticle cluster and the Brownian motion of the nanoparticles. The thermal conductivity property was augmented by 5.35%, 11.47%, and 19.12% at 0.15, 0.3, and 0.45 wt. % of MWCNTs loading at room temperature respectively.

**Fig. 14:**
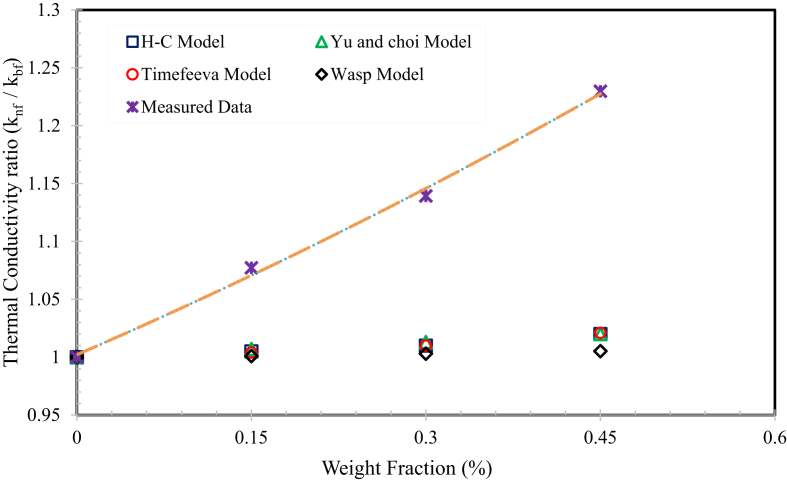
Thermal conductivity ratio comparison with present correlations.

Fig. 14 represents the measured values of the “*k*” of the nanofluids at 30 °C as a function of weight fraction, together with Hamilton-Crosser model [52], Wasp model [53], Yu and Choi [54] Model, and Timefeeva [55] model and predictions. The comparison values definitely show that the measured values are greatly higher than the predicted values for all the nanofluids. This was owing to the difference in nanomaterial size, a method of preparation, nanomaterial distribution, nanomaterial cluster and interfacial layer between the nanoparticle and the base fluid. Owing to the absence of an appropriate and suitable correlation to predict the ratio of thermal conductivity of the MWCNTs nanofluid of solar glycol – water. The experimentally measured trend lines are described in the graph and the experimental values are the finest fitting with polynomial functions. Therefore, the proposed correlation, with an accuracy of R^2^ = 0.9967.

$$\text{The ​ratio ​of ​thermal ​conductivity(}k_{r}\text{)}=\frac{k_{nf}}{k_{bf}}=\text{ ​}0.1465\phi^{2}\text{ ​}+\text{ ​}0.435\phi \text{ ​}+\text{ ​}1.0022$$

### 3.3 Electrical conductivity enhancement of nanofluids

**Fig. 15:**
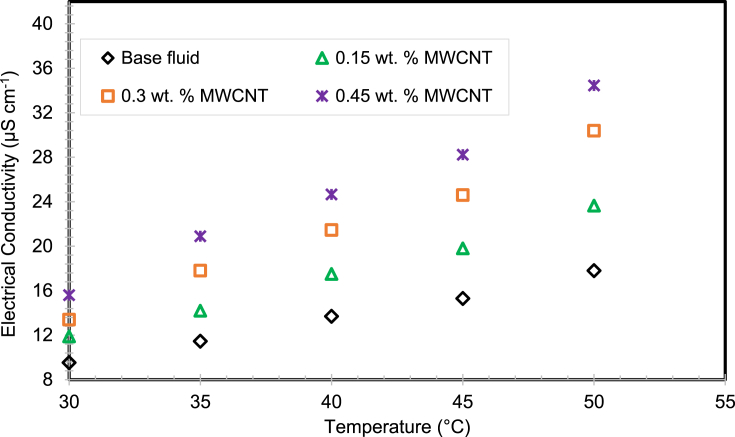
The EC of MWCNT nanofluids.

**Fig. 16:**
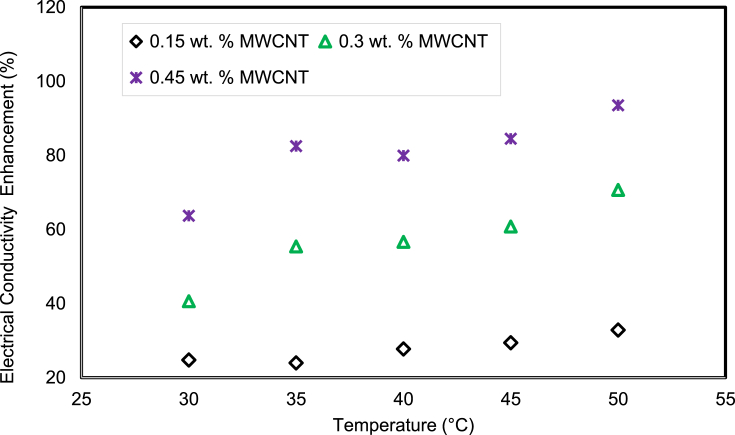
EC augmentation of the MWCNTs based nanofluid with temperature.

The EC of the virgin base fluid increased when the nanomaterials were seeded into the H_2_O/SG mixture. Fig. 15 displays the EC variation of H_2_O/SG blended nanofluids with various nanomaterial weight percentage and temperatures between 30 ^ο^C and 50 °C in steps of 5 °C. It is observed from the measured results that the EC of MWCNTs added nanofluids is greater than that of the H_2_O/SG mixture. Similarly, the augmentation in EC of nanofluids is a combined function of weight fraction of nanomaterials and the seeding temperature. Maximum EC enhancement of 93.54% was achieved with 0.45% weight percentage at 50 °C nanofluid temperature related to the H_2_O/SG blend as explained in Fig. 16.

## 4 Conclusions

The present research, stability, density, viscosity, electrical conductivity and the thermal conductivity of MWCNTs based nanofluids prepared with nanomaterial weight fractions of 0.15, 0.3, & 0.45 wt. % was measured experimentally.

• UV-Vis, SEM, and sedimentation analysis indicate that the prepared MWCNTs based nanofluids have better homogenous stability for a period of more than 2 months.
• The density of the studied nanofluids enhances with a surge in MWCNTs concentration. The calculated data display evident deviance from the projected values of the Pak and Cho models. The deviance rises with an upsurge in the MWCNT concentration.
• The higher thermal conductivity augmentation of 19.12% was achieved for 0.45 wt. % MWCNTs loading at room temperature owing to the kinetics of nanomaterial accumulation and fluid layering. Therefore a significant enhancement in dynamic viscosity with an increase in nanomaterial weight fractions.
• The EC gets augmented in a nonlinear way with respect to the seeding of MWCNTs. Nevertheless, the EC of SG – water mixture based nanofluids is augmented linearly with increase in temperature. At 0.45 (in wt. %) weight fraction, the electrical conductivity (σ) of the nanofluid is improved by 93.54% at a temperature of 50 °C.
• Correlations were established from the studies for estimating the thermal conductivity and the viscosity of the SG – water mixture based nanofluid. The H_2_O/SG blended based CNTs nanofluids can be recommended for the heat transfer fluids in engine cooling, electronic cooling, solar thermal, cooling in machining and other process industrial applications.

## Declarations

### Author contribution statement

P. Ganesh Kumar: Conceived and designed the experiments; Performed the experiments; Wrote the paper.

D. Sakthivadivel: Performed the experiments; Analyzed and interpreted the data.

M. Meikandan: Contributed reagents, materials, analysis tools or data; Wrote the paper.

V. S. Vigneswaran: Performed the experiments; Contributed reagents, materials, analysis tools or data.

R. Velraj: Conceived and designed the experiments; Wrote the paper.

### Funding statement

This research did not receive any specific grant from funding agencies in the public, commercial, or not-for-profit sectors.

### Competing interest statement

The authors declare no conflict of interest.

### Additional information

No additional information is available for this paper.
